# Comparison between computer-guided and conventional paper-based insulin infusion protocols in the treatment of acute hyperglycemic syndromes: an observational study

**DOI:** 10.62675/2965-2774.20250164

**Published:** 2025-05-06

**Authors:** Laura Andrade Mesquita, Marcos Tadashi Kakitani Toyoshima, Carolina Luz Silva, Alina Coutinho Rodrigues Feitosa

**Affiliations:** 1 Escola Bahiana de Medicina e Saúde Pública Salvador BA Brazil Escola Bahiana de Medicina e Saúde Pública - Salvador (BA), Brazil.; 2 Hospital das Clínicas Faculdade de Medicina Universidade de São Paulo São Paulo SP Brazil Instituto do Câncer Octávio Frias de Oliveira, Hospital das Clínicas, Faculdade de Medicina, Universidade de São Paulo - São Paulo (SP), Brazil.; 3 Hospital Santa Izabel Salvador BA Brazil Hospital Santa Izabel - Salvador (BA), Brazil.

Acute hyperglycemic syndrome (AHS), including diabetic ketoacidosis (DKA) and a hyperosmolar hyperglycemic state (HHS), is a critical emergency requiring prompt and effective management. Intravenous insulin therapy is the cornerstone of treatment. Electronic glucose management systems (eGMSs) are increasingly used in treating AHS,^(1)^demonstrating positive outcomes such as the resolution of AHS^(2,3)^and a reduction in the incidence of hypoglycemia.^(2,4,5)^ InsulinAPP-UTI®, a Brazilian eGMS, is available in Portuguese, English, and Spanish and was initially developed for managing hospital hyperglycemia in critical patients;^(6,7)^however, its potential benefits in managing AHS are worth exploring.

This retrospective cohort study compared the efficacy and safety of InsulinAPP-UTI® (eGMS group) with those of a conventional paper-based protocol (PP group) in managing AHS.

We included adult patients with AHS, as per the American Diabetes Association (ADA) criteria,^(8)^ treated with intravenous insulin therapy. The InsulinAPP-UTI® was implemented in June 2020, allowing comparisons between the preimplementation (May 2019 - May 2020) and postimplementation (July 2020 - July 2021) periods. June 2020 was considered a transition period. The PP followed the ADA recommendations for insulin therapy.^(8)^ InsulinAPP-UTI® insulin dose calculations are based on current blood glucose levels and their variation over time.^(7)^ The study was approved by the Ethics Committee for National Research (CAAE: 59667622.0.0000.5520).

The primary outcome was the resolution of AHS, defined by normalization of blood glucose levels and resolution of acidosis or hyperosmolality.^(8)^ Secondary outcomes included the length of hospital stay, mortality, time to resolution of AHS, number of patients with hypoglycemic events (< 70mg/dL), hypokalemia (< 3.3mEq/L), intensive care unit admission rate, and reintroduction of intravenous insulin after suspension. The frequency of blood tests was similar in both groups, including capillary blood glucose every one to two hours and monitoring of electrolytes (sodium and potassium) every two to four hours until the patient was stable.^(8)^

Statistical analysis was performed via SPSS version 14.0 (Chicago, USA), with p values < 0.05 considered significant.

**Figure 1:**
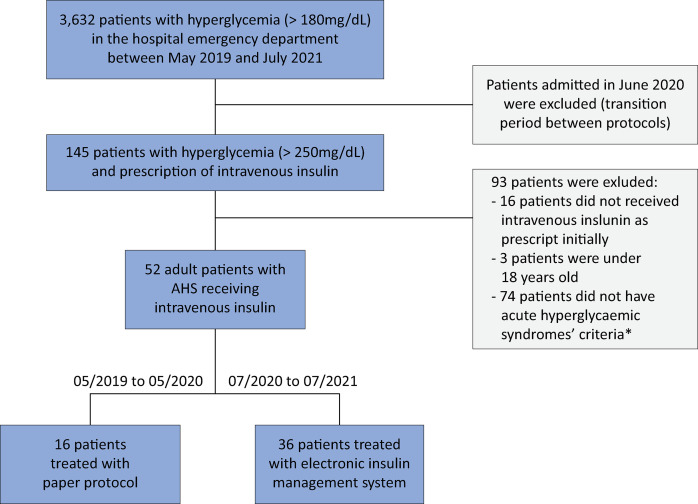
Patient selection process. AHS - acute hyperglycemic syndrome. *Acute hyperglycemic syndrome criteria: diabetic ketoacidosis: glucose > 250mg/dL, metabolic acidosis, and positive ketonemia or hyperosmolar hyperglycemic state: glucose > 600mg/dL and seric osmolality > 320mOsm/kg.

Among the 3,632 patients screened, 52 met the inclusion criteria: 16 in the PP group and 36 in the eGMS group (Figure 1). Baseline characteristics, including age, sex, and body mass index, were similar between the groups. However, the eGMS group had a greater prevalence of type 2 diabetes (T2D; 93.1% *versus* 50.0%, p < 0.05). Only the eGMS group included patients with COVID-19. Corticosteroid use at hospital admission and the patient severity score (APACHE II) were similar in both groups.

The eGMS group demonstrated 80.5% resolution of AHS, and the PP group demonstrated 68.7% resolution. However, the difference was not statistically significant. Similarly, no significant differences were observed in mortality, hypoglycemic events, or time to achieve AHS resolution (Table 1).

**Table 1:** Outcomes of patients with acute hyperglycemic syndrome treated with a paper-based protocol and an electronic glucose management system

Variable	Paper-based protocol	eGMS	p value	OR (95%CI)
	(n = 16)	(n = 36)		
AHS resolution during hospitalization	11 (68.7)	29 (80.5)	0.478	0.53 (0.13 - 2.03)
Time to AHS resolution (hours)	25.5 (7.5 - 45.5)	27.8 (8.5 - 87.5)	0.261	
Mortality	4 (25)	11 (30.5)	0.752	0.75 (0.19 - 2.8)
Incidence of hypoglycemia (< 70mg/dL)	10 (62.5)	21 (58.3)	0.777	1.19 (0.35 - 3.99)
Incidence of hypokalemia (< 3.3mEq/L)	6 (37.5)	11 (30.5)	0.622	1.36 (0.39 - 4.69)
ICU admission	15 (93.8)	26 (72.2)	0.140	5.76 (0.67 - 49.60)
Reintroduction of intravenous insulin after suspension	4 (25)	9 (25)	1.000	1.00 (0.25 - 3.89)
Corticosteroid usage in hospital admission	2 (12.5)	5 (13.9)	1.000	0.88 (0.15 - 5.13)
APACHE II score	13.5 (7.5 - 17.7)	15.0 (9.0 - 24.3)	0.290	

eGMS - electronic glucose management system; OR - odds ratio; 95%CI - 95% confidence interval; AHS - acute hyperglycemic syndromes; ICU - intensive care unit; APACHE II - Acute Physiology and Chronic Health Evaluation II. p values < 0.05 were considered statistically significant. Data were presented in odds ratio and 95% confidence interval. Quantitative variables with nonnormal distribution were expressed as median and interquartile range, and Mann–Whitney test was performed. Qualitative variables were described in absolute value and percentage, and Fisher’s exact test was performed.

The COVID-19 pandemic has led to the use of high doses of corticosteroids, cytokine storms, poorer COVID-19 outcomes in preexisting diabetic mellitus patients and overload of the health care system,^(9)^ which could also explain the high mortality in the eGMS group, despite greater AHS resolution.

The study´s limitations, including the retrospective and pre- and postintervention study design, small sample size and the impact of confounding factors such as the COVID-19 pandemic,^(9, 10)^and the higher prevalence of T2D in the eGMS group, limit the generalizability of the findings. These factors may have introduced biases that influenced the outcomes.

While the study did not find significant differences between InsulinAPP-UTI® and the conventional paper protocol in resolving AHS, the results suggest that InsulinAPP-UTI® may offer comparable efficacy. Further prospective studies with larger, more homogeneous samples are necessary to validate the efficacy of InsulinAPP-UTI® in managing AHS and to assess its long-term impact on clinical outcomes.
